# “Architects of Their Own Brain.” Social Impact of an Intervention Study for the Prevention of Gender-Based Violence in Adolescence

**DOI:** 10.3389/fpsyg.2019.03070

**Published:** 2020-02-06

**Authors:** Sandra Racionero-Plaza, Leire Ugalde, Guiomar Merodio, Nerea Gutiérrez-Fernández

**Affiliations:** ^1^Department of Sociology, University of Barcelona, Barcelona, Spain; ^2^Department of Didactics and School Organization, University of the Basque Country, Leioa, Spain; ^3^Department of Education, Nebrija University, Madrid, Spain; ^4^Faculty of Psychology and Education, University of Deusto, Bilbao, Spain

**Keywords:** gender violence prevention, adolescence, intervention research, social impact, dominant coercive discourse, mental models of attraction, memory, peer group

## Abstract

Research in psychology has evidenced both the prevalence of gender-based violence among youth worldwide and the negative impacts that such violence has on the victims’ mental and physical health. Neuroscience has proven that violent intimate relationships harm the brain, while very simple social experiences can change the brain architecture in positive directions. Also, interventions that have been demonstrated to be successful in preventing and responding to gender violence in adolescence have been informed by psychology. This article reviews the social impact of psychology in the field of teen gender violence and then reports on the potential social impact achieved by an intervention study consisting of seven interventions framed by the research line on the preventive socialization of gender violence. The program was addressed to 15- and 16-year-old adolescents and focused on supporting free reconstruction of mental and affective models of attractiveness via critical analysis of the *dominant coercive discourse*, which links attraction to violence. The communicative methodology involved working with an Advisory Committee from the beginning of the study, as well as continuous dialog between the researchers and the participants, which was used to refine subsequent interventions. The results show that the program contributed to raising participants’ critical consciousness regarding the dominant coercive discourse in their life, provided the participant subjects with cognitive tools to better understand their own and others’ sexual-affective thinking, emotions, and behaviors, in favor of rejecting violence, and supported the modification of female adolescents’ sexual preferences for different types of men. Importantly, the findings also indicate that the interventions aided some participants’ use of the knowledge gained in the project to help their friends and communities in reflecting upon coercive patterns of sexual attraction, the quality of their intimate relationships, and the different effects of sexual violence and toxic relationships on health. Some individuals reported leaving toxic relationships after the interventions. This intervention research illustrates Santiago Ramón y Cajal’s metaphor, employed to explain plasticity: that every person, if s/he decides it, can be the architect of her or his own brain. With evidence-based cognitive tools within the reach of every adolescent, and upon individual free choice for transformation, a new sexual-affective socialization free from violence is possible.

## Introduction: Social Impact of Psychological Research on Gender Violence

The research and innovation framework defined by the European Union, Horizon 2020, leaves no doubt about the need for science to be carried out with and for society (European Commission, 2014). Research must respond to social challenges by helping to create better societies (European Commission, 2018) and improving the lives of all citizens (Flecha et al., 2015). The social problems to be addressed by researchers are set by citizens and global institutions. The United Nations’ 2030 Agenda in relation to sustainable development has shared 17 goals that are central to promoting prosperity and protecting the planet and which must be assumed by all countries, poor, rich, and middle-income. One of those goals, number five, focuses on gender equality, and gender violence is identified as one central target for making the goal real. The United Nations (2019) states that in the last 12 months, one out of five women between 15 and 49 years old have suffered physical or sexual violence by their partners and that there are currently 49 countries in which there is no law to protect these women from this type of violence. Society is concerned about this problem. In an investigation conducted to identify the relevance of research aims through the voices of citizenship in social networks, researchers found that “violence against women” was one of the most used keywords by users in social networks and in online resources (Cabré et al., 2017). Overcoming gender violence is a global social concern that urges evidence-based solutions.

### Contributions of Psychology to Tackling Gender Violence

Research in psychology has been and continues to be key to helping us tackle gender violence worldwide, in terms of establishing its prevalence and informing its prevention. Indeed, thanks to the evidence provided by psychological research, we know that gender violence is a global problem (United Nations Statistics Division, 2015) that is suffered by women of different ages (Stöckl et al., 2014) from all socio-economic backgrounds and that occurs both in stable and sporadic relationships (Trygged et al., 2014; Bay-Cheng and Bruns, 2016; Puigvert et al., 2019). Several studies in psychology have shown a non-negligible prevalence of gender violence in the sexual-affective relationships of young people (Blattner et al., 2015; World Health Organization, 2015; Ybarra et al., 2016; Fernández de Juan and Florez Madan, 2018). Moreover, one of the first studies, which analyzed data about the prevalence of Intimate Partner Violence (IPV) among adolescents and young women in nine countries, found that young women have a higher risk of experiencing IPV than older women (Stöckl et al., 2014). Stöckl et al. (2014) reported a prevalence of around 50 percent or higher in most sites, referring to the proportion of young women (between 15 and 24-years old) who had ever suffered IPV. The magnitude of this problem in adolescents is considered alarming (Lundgren and Amin, 2015) and is generating increasing concern among practitioners, scientists, policy-makers, and society as a whole (Barter, 2007; De Koker et al., 2014) given the devastating effects that research in psychology has shown that gender violence can have on the health and positive development of the victims (De Koker et al., 2014).

At the psychological level, research has reported a higher risk of the victims experiencing symptoms of depression and anxiety (Ackard et al., 2007; Exner-Cortens et al., 2013) and suicidal ideation (Ely et al., 2011; Exner-Cortens et al., 2013), among other disorders. Unhealthy behaviors such as using tobacco, drugs, and alcohol (Roberts et al., 2003; Exner-Cortens et al., 2013) or suffering from eating disorders (Ackard and Neumark-Sztainer, 2002; Sharp and Keyton, 2016) are found to be among the most common consequences in terms of habit disorders. At the educational level, there is evidence that demonstrates a higher risk of gender-based violence victimization during college among those who have suffered from IPV (Smith et al., 2003) and a decline in academic performance or increase in the dropout rate (Banyard and Cross, 2008; Holmes and Sher, 2013). This evidence from psychology provides strong arguments for the development of interventions that prevent gender violence among adolescents and youth, supporting the avoidance of future revictimization.

Psychological research has also been central in pointing out the risk factors for gender violence victimization, namely, among others, peer influence, substance abuse, psychological adjustment, and attitudes toward violence (Leen et al., 2013). Other interdisciplinary research has shown that socialization into a *dominant coercive discourse* (Puigvert and Flecha, 2018), where males with violent attitudes and behaviors are presented as more sexually attractive, is also one cause for gender violence among teenagers (Gómez, 2015). Such dominant coercive discourse is spread widely by the media, peer groups, and other socialization agents. Nevertheless, research in psychology, sociology and other social sciences has also evidenced that new interactions and social experiences can drive the learning of new attraction patterns that are able to weaken the link between violence and attractiveness and the development new mental and affective models where sexual attraction is associated with dialog and respect (Gómez, 2015; Racionero-Plaza et al., 2018). This evidence supports the relevance for the design and implementation of interventions and programs that provide new opportunities for youth to revise their sexual-affective mental and affective models and to reconstruct them in the direction of avoiding violent intimate relationships, if the adolescents freely decide to do so. The program reported in this article responds to this need.

Psychology has also advanced knowledge that has helped in the design of more effective preventive actions. Psychological research has shown that preventive interventions, to be successful, have to take place in the environments where adolescents socialize, the school and the community mostly, and involve key adults, such as teachers, parents, or other community members (De Koker et al., 2014). Since the responsibility to combat violence against women is collective, the response must be collective too (Wagner and Magnusson, 2005). Thus, studies suggest that, among others, during childhood and adolescence, educational settings are ideal spaces in which to act preventively (Ozer, 2006; Lundgren and Amin, 2015; Theimann, 2016; Nakray, 2018; Solís Dominguez and Martínez Lozano, 2018), including by transforming hostile environments created by peers in high schools where sexual harassment happens (Fineran and Bennett, 1999).

Research in psychology has also shed light on the developmental stage at which to engage in preventive work on gender violence so as to raise its effectiveness (De Koker et al., 2014). This intervention period is adolescence. Adolescence and pre-adolescence is the time when the first sexual-affective relationships are established for many teens, and those first experiences will become the basis for subsequent healthy or toxic relationships (Stöckl et al., 2014; Gómez, 2015). Also, it is in adolescence when gender role differentiation strengthens, changing the way of acting in sexual-affective relationships, which represents a great opportunity to work on the promotion of attitudes to prevent IPV (Lundgren and Amin, 2015).

### Social Impact of Psychology in the Area of Gender Violence

On the basis of all of the scientific evidence in psychology accumulated throughout time in relation to gender violence, a number of interventions and programs have been designed to tackle gender violence among adolescents and youth, with many proving their efficacy in dimensions associated with the problem.

Grounded on the evidence provided by psychology and other social sciences on the importance of educational settings as contexts for preventive action, diverse school-based dating violence prevention programs have shown proof of effectiveness in preventing perpetration and/or victimization of dating violence (De Koker et al., 2014; Lundgren and Amin, 2015). Three well-known programs are a good example of this. The *Safe Dates Project* (Foshee et al., 2005) is one of them. Including 10 classroom sessions taught by health and physical education teachers, an informational poster of the taught content, and a play performed by students, the program reported less psychological, moderate physical, and sexual dating perpetration and reduced moderate-physical dating violence victimization in adolescents who took part in it (Foshee et al., 2005). Secondly, *The Four R* program, which integrates 21 lessons focused on healthy relationships, sexual health, and substance use prevention into the curriculum, obtained a reduction of physical dating violence among participants (Wolfe et al., 2009). Third, the importance of focusing on all school contexts was observed in the *Shifting Boundaries* intervention (Taylor et al., 2013, 2015). This program offers a classroom intervention, a building intervention, and a combined intervention or neither intervention options, and it was found that the building only and the combined intervention were effective in reducing sexual violence perpetration and victimization (Taylor et al., 2013, 2015).

Research has also shown that it is important that intervention programs for the prevention of gender violence among youth maintain the positive effects of the interventions over time. In this regard, studies have shown that obtaining behavioral changes in relationships, skills, and attitudes seems to be a factor that can help promote sustainability (Leen et al., 2013). Therefore, it is not enough for intervention programs to focus on decreasing dating violence; it is necessary as well to promote healthy relationships in order to sustain change over time (Miller et al., 2015), and friends, teachers, parents, or people from the community can help in achieving this; strong and positive interpersonal relationships are crucial for adolescents’ healthy development and well-being. Along these lines, research has provided evidence that bystander attitudes from witnesses are essential to stop violent behaviors and set the basis to create environments where healthy relationships are prioritized. *TakeCARE* is a video bystander program designed to promote bystander behavior in situations where violent relationships and sexual violence happens in high schools (Sargent et al., 2017). The adolescents who watched the video were involved in more bystander behaviors than the students who did not watch it Sargent et al. (2017). This behavior needs to be extended to other contexts in which adolescents develop to achieve even better results. The intervention that Miller et al. (2013) carried out, training athletic coaches to integrate prevention messages in the activities that they guide, is a good example of this. This program achieved a lower level of negative bystander behaviors and less prevalence of dating violence in athletes involved in the intervention (Miller et al., 2013). These evidence-based programs show the importance of creating a sense of community and of feeling emotionally safe to promote healthier relationships. Different approaches can be taken to achieve such results. Dialog in support groups is a successful option that was evidenced in the *Expect Respect* teen violence prevention program (Ball et al., 2009). The promotion of positive communication between young people and its benefits were also reported by the *Healthy Relationship Campaign*, which, through the use of text messages, developed a sense of community and promoted healthy relationships between the adolescents who took part in the program (Guillot-Wright et al., 2018). To maintain this climate of respect, the prevention of peer aggressive behaviors needs to be developed not only in face-to-face environments but also inside social networks, as the *DARSI* program has achieved (Carrascosa et al., 2019).

Psychology has also shown that building safe environments and promoting healthy relationships is especially important in the case of adolescents who are in a situation of vulnerability, and a number of programs have been designed based on such knowledge. To prevent dating violence against pregnant and parenting teens, an adaptation of the *Safe Date* program curriculum was carried out that obtained a reduction of incidence among participants (Herman and Waterhouse, 2014). Other successful interventions such as *Love 2*, addressed to low-income and high-risk youth (Antle et al., 2011), or the *Youth Relationship Project*, for teens with histories of maltreatment (Wolfe et al., 2003), have been implemented with good results. Programs that have taken into account differences in dating history and in history of dating violence victimization and perpetration, such as the *Teen Choices* online program, have achieved successful outcomes (Levesque et al., 2016).

Along the lines of emphasizing interventions with more vulnerable adolescents, preventive programs tackling the prevention of gender violence revictimization among youth becomes a priority. In this regard, research has indicated the importance of providing tools that enable youth to be more critical about violence (Aiello et al., 2018) and, starting from the power of the *dominant coercive discourse* in their sexual-affective socialization, to revise and modify attraction toward men with dominant and aggressive behavior (Gómez, 2015). Work on autobiographical memories of violent sexual-affective relationships can play an essential role in this sense, as this type of memory influences prospective thinking and behavior (Williams et al., 2008; Klein et al., 2010), emotional well-being, and overall health (Rubin, 2010). Interventions that aid young women in revising and reconstructing memories of violent sexual-affective relationships in positive directions, including the transformation of the emotions induced by the recall toward feelings of rejection, are grounded in the evidence of the malleability of autobiographical memories (Cohen and Conway, 2008) via specific learning experiences (Stone et al., 2010; Hirst and Rajaram, 2014). Such memory-based interventions have already proven promising for the prevention of gender-based violence revictimization among young women (Racionero-Plaza et al., 2018).

All of the aforementioned prevention programs have been informed by particular research findings from psychology and other social sciences on gender violence among teens and have made it possible for adolescents to engage in specific transformations of their sexual-affective thought and behavior in ways that can protect them from experiencing violence in their intimate relationships. The case of the MEMO4LOVE project, the intervention research reported in this article, builds upon the various research projects on the topic of preventive socialization of gender violence (Valls et al., 2008; Gómez, 2015; Puigvert, 2016; Puigvert et al., 2019), which have developed a strong body of evidence on the need to tackle the dominant coercive discourse in society to help youth escape from the social imposition of attraction models that link desire with violence and aggressiveness. The intervention program in the project adds to the current knowledge on prevention strategies for adolescents by focusing on the dominant coercive discourse, being a group-level intervention and school-based. The program is part of a group of interventions known as a ‘dialogic model of conflict prevention and resolution.’

## Materials and Methods

### Participants

The sample of participants in the MEMO4LOVE project comprised 126 adolescents attending three different high schools in Barcelona (Spain). This number fluctuated slightly throughout the project, as not all students in all classrooms attended every intervention. The three high schools constituted the experimental groups of the study, and the program of interventions for preventive socialization of gender violence was implemented over a period of 6 months (January–June 2019) in these schools. The interventions took place in the 4^*th*^ grade of Compulsory Secondary Education. The age of the participants mostly ranged from 15 to 16 years. Two of the high schools were public, and one was semi-private. The class groups in all three high schools were highly diverse ethnically and culturally, and mostly had mid-low and low SES. Participants in the communicative focus groups and interviews were randomly selected females and males, and the questionnaires evaluating every intervention were answered by all students who had participated in the project.

#### Inclusion Criteria

Participants in the interventions and in the data collection were all students, females and males, enrolled in the 4^*th*^ grades at every high school involved in the project, whose parents had given written informed consent for their participation in the study, as well as they themselves having provided written informed assent. No remuneration was given either to the adolescents or to the high schools for their participation in the study. The significant participant involvement in the project was supported by the commitment of the three high schools to equipping their students with evidence-based tools to prevent gender-based violence victimization or revictimization; no other activities on the same topic were taking place when the MEMO4LOVE interventions were implemented.

### Material and Procedure

This study followed all ethical standards for research involving human participants in the Declaration of Helsinki (World Medical Association [WMA], 2013) and from Horizon 2020 (European Commission). Before participant subjects became involved in the study, the researchers fully informed the principals and teachers in the high schools, the parents and tutors of the adolescents, and the adolescents themselves about the project. In those informative sessions, all of them could ask questions of the researchers. The high schools received an informative letter sharing details of the study. Given that participants were under age 18, their parents and tutors completed written Informed Consent forms. Adolescent students were also informed orally via talks in their classes, had time to read the assent form and to ask questions of the researchers, and completed written Informed Assent forms afterward. Explanations were given by the researchers when necessary to the principals, teachers, parents, tutors, and students at any time before and during the interventions. The information provided in the consent and assent forms explained the objective of the study, the voluntary nature of participation, the possibility to withdraw from the study at any time, the procedure to collect the data, the materials and measures to be used, and the anonymity and privacy statement. Codes were always employed in the quantitative data collection, and pseudonyms have always been used for quotations. The letter for the high schools also included this information. The Research Ethics Committee of the Virgen de la Macarena and Virgen del Rocío Hospitals (Government of Andalusia) revised and fully approved the study.

The entire project was conducted using the communicative methodology of research (Soller-Gallart, 2017). This election was made because the communicative methodology had proven efficacy in raising the social impact of research (Flecha et al., 2015), including the social impact of research on gender violence (Gómez González, 2019). The employment of this methodology in the project implied not only the use of communicative data-collection techniques and egalitarian interaction between the researchers and the research subjects but also the incorporation of some aspects of communicative organization. Among those aspects, of note was the constitution of an Advisory Council (AC) from the very beginning of the project. This AC was composed of representatives from two NGOs dedicated to education, one professional working with adolescents in the mental health field, one high school teacher, and two researchers in the field of gender violence prevention and response, one from the area of feminist studies and the other from the area of masculinity studies. The AC gave feedback on the program of interventions before its start, and members of the AC were asked for their inputs at certain moments within the project to make adjustments in the development of the program of interventions to better meet the schools’ and adolescents’ needs. Additionally, following the communicative paradigm, the questionnaires, surveys, and interview guidelines elaborated on the project, and the translation of other existing instruments was validated with adolescents different from the ones who later engaged in the program but of the same age and SES. This validation ended up introducing modifications in the translations of some instruments and language edits in one of the questionnaires and in the interview protocols. This communicative process of refinement was important to better tackle the object of the study and increase the likelihood of potential social impact. Also, the employment of the communicative methodology facilitated the finding of solutions to the difficulties and challenges that emerged during the intervention research in natural contexts, mostly changes in the schedule of interventions that resulted from needs and unforeseen circumstances in the high schools. The egalitarian dialog between the research team and members from the high schools and the high ethical standards of the same communicative methodology were very effective tools for tackling the challenges of the research process.

### Intervention

**FIGURE 1:**
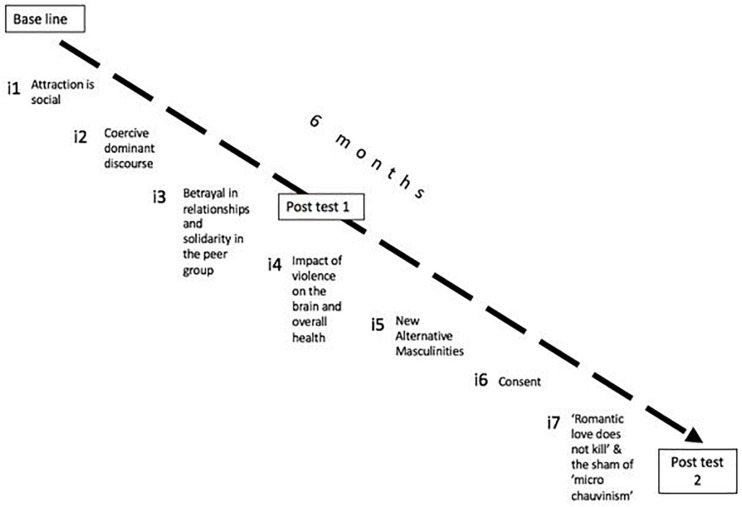
Overview of the interventions of preventive socialization of gender violence implemented in the project and the timings of measurements.

The MEMO4LOVE research project involved the design of a program of seven interventions (see Figure 1) for the preventive socialization of gender violence. All interventions were grounded in evidence collected from the research line of preventive socialization of gender violence developed at the Community of Researchers on Excellence for All (CREA) and also built upon other scientific evidence in the social and natural sciences about gender violence and toxic relationships. Every intervention lasted for an hour and took place during the school day, in the natural setting, the classroom, and in the natural group, the class group.

Interventions 2, 3, 4, 5, and 6 followed the format of lecturing by a researcher from the project in which scientific evidence on the topic of the session was shared, followed by a dialog with the whole group. The lectures were always supported by a PowerPoint presentation. The dialog with the class group was guided by two or three general questions but was open to taking new questions from the adolescents. Intervention 1 involved, instead of lecturing, the screening of a video of a lecture given by Professor Gómez (2004) on the social nature of love and attraction; this researcher was a leader in the field of research of preventive socialization of gender violence. Intervention 7 involved reading two brief texts published in *Diario Feminista* (Joanpere, 2017; Duque, 2018) and discussing them, following some guiding questions. One of the texts dealt with the idea that lacks scientific support but is much promulgated that ‘love kills,’ and the other text questioned another disseminated assumption in Spain on ‘micro chauvinism.’ Intervention 2 focused on the dominant coercive discourse; intervention 3 dealt with infidelity as a behavior that is submissive to the dominant coercive discourse and the role played by friends and peers in this circumstance. Intervention 4 shared scientific evidence in psychology, psychiatry, neuroscience, genetics, and medicine on the negative impact of toxic relationships on neural connections and brain architecture, even leading to neural death, as well as involving discussion of the evidence on the positive effects of quality close relationships on the brain and overall health. Intervention 5 presented the New Alternative Masculinities (NAM) as masculinities that are egalitarian and attractive (Flecha et al., 2013); and intervention 6 raised the important topic of consent in sexual-affective relationships. All interventions had the definitory characteristic of sharing with adolescents the scientific evidence on the topics discussed in every intervention, thus making real article 27 of the Universal Declaration of Human Rights United Nations (1948), which establishes all humans’ right to access scientific knowledge and to participate in it. Also, in all interventions, the researchers linked the conceptual knowledge to everyday experiences of adolescents, to the TV series that many of them watch, the singers that many of them follow, the songs that are famous among them, etc., as illustrations of the dominant coercive discourse as well as to shed light on alternative sexual-affective models grounded in both equity and attraction. All interventions started from the hypothesis of brain plasticity and were designed and approached from the perspective of socioneuroscience, a line of research that understands that social interactions and experience shape neural wiring. While the dominant coercive discourse imposes a neural wiring that enslaves sexual-affective patterns of attraction, individuals, with the right stimuli and if they decide to do so, can raise their critical awareness about the dominant coercive discourse, revise such mental models, and rewire their brains to break free from such discourse.

Figure 1 presents an overview of the seven interventions implemented in the project and the timings of measurements.

### Measures and Instruments

The intervention project followed a longitudinal design of repeated measures. Quantitative and qualitative data were collected before the first intervention, as a baseline measurement. Once the program began, measurement was conducted for a second time (post-test 1) after intervention 3 and for a third and final time (post-test 2) when all interventions finished, that is, after intervention 7. The quantitative measures involved surveys and questionnaires elaborated for the project and other standardized instruments; they dealt with peer interactions’ influence on gender violence, with attitudes toward violence in sexual-affective relationships, and with autobiographical memories of violent relationships. Qualitative data collection involved communicative focus groups and in-depth interviews with the participant subjects. Also, a survey was used to collect adolescents’ perceptions of the utility and impact of every intervention. This written survey was administered immediately after every intervention.

In this manuscript, we report data from (a) 10 communicative focus groups that were conducted in the three high schools, in post-test 2 (month 6 and month 7), to specifically examine the impact of the project’s interventions on adolescents’ mental and affective models related to sexual attraction and gender violence, as well as upon their and their peers’ behavior, and (b) the surveys that evaluated adolescents’ perception about the value of every intervention. Regarding the focus groups, out of the 10, four were with girls only, five were with girls and boys, and one was with boys only. The communicative focus groups are natural groups that construct a collective interpretation of reality through egalitarian dialog among all members, who have a nexus. In regard to the evaluation surveys, they were administered to the 126 adolescents in the project and involved four questions that focused on assessing: the utility of the intervention, whether there was different thinking after the intervention (if any), where there was better understanding, after the intervention, of things that happen to the adolescent or to his/her friends and other people (if any), and whether there was better ability, after the intervention, to help friends and others (if any). As for the first question, it read, “How useful was this session for reflecting about your feelings and relationships?,” and responses were given on a five-point Likert scale ranging from not at all (1) to totally useful (5). The second question posed was, “Do you think that you now think differently about the topics that have been discussed?,” and possible answers were given on a three-point Likert scale ranging from very different (1) to the same (3). The third question read, “Do you think that you now understand better some things that happen to you or to your friends?,” with possible responses given on a three-point Likert scale ranging from much better (1) to the same (3). The fourth question was, “Do you think that you can now better help your friends?,” and answers were given on a three-point Likert scale ranging from much better (1) to the same (3).

### Data Analysis

#### Coding of Qualitative Data

The qualitative data was analyzed to identify the perceptions of adolescents regarding the impact of the program upon their mental and affective models of sexual-affective attraction and attitudes on gender violence prevention and response. Taking into account that psychological research has indicated that are gains that contribute to preventing the risk of gender violence victimization and the reduction of such, we elaborated a coding scheme for the analysis of the data that consisted of the following categories: (1) *Consciousness about the dominant coercive discourse in one’s life*: adolescents’ consciousness about the presence and influence of the dominant coercive discourse in their life and in that of their peers, friends, and other people they know. This consciousness can also occur via recovering autobiographical memories of experiences influenced by the dominant coercive discourse. (2) *Change in mental models of attraction*: change in thoughts about who is attractive (and successful) and why in line with emptying violent masculine models of attraction; (3) *Change in affective models of attraction*: change in emotions and feelings of attraction in line with feeling less attraction toward and rejection of males with aggressive attitudes and behaviors and more attraction toward egalitarian men; (4) *Taking a stand and help-giving*: providing help to friends, peers and other people in the community using the scientific knowledge gained in the interventions; this usually involves taking a stand against sexual-affective violence; (5) *Impact*: interventions with most impact for the adolescents and why.

This coding scheme was open enough to incorporate new emerging codes from the data. This bottom-up approach provided a new category of analysis named “new cognitive tools” (6), which are tools with which to analyze one’s sexual-affective life and that of one’s friends, family, and community members.

The quantitative data from the surveys evaluating the utility of the interventions were analyzed per intervention, and comparisons between interventions were performed to complement the results of the qualitative analyses about interventions with the most impact.

## Results

The analysis of the data from the communicative focus groups and interviews in the second post-test, and quantitative data from the evaluation questionnaires administered after every intervention showed that: (a) the program contributed to raising participants’ critical consciousness regarding the presence of the dominant coercive discourse in society and in their life, (b) the interventions provided cognitive tools to the adolescents to better analyze their own and other’s sexual-affective thinking, emotions, and behavior, in ways that favored rejection of violent intimate relationships, (c) the program supported the modification of female adolescents’ sexual preferences for different types of men; (d) some participants used the cognitive tools learned in the interventions to help friends and community members in reflecting upon patterns of sexual attraction, the quality of their intimate relationships, and their different effects on health. Such advice led some of those who received help to become aware of the type of relationship that they had and its health implications, with some of them even deciding to break with the violent sexual-affective relationship they had at that moment.

In what follows, each of these main results is presented accompanied by quotations from the focus groups and interviews and, in some cases, with complementary quantitative data.

### Recovering Autobiographical Memories and Raising Consciousness Regarding the Influence of the Dominant Coercive Discourse in One’s Life

Interventions supported adolescents’ critical awareness of the influence of the dominant coercive discourse in their life, present and past, promoting recovering memories of social experiences where discourse that links males with aggressive attitudes and behavior with attractiveness was present. In one of the focus groups, Ana explained that the interventions made her recall some movies that she had watched in the past. She emphasized that she experienced this particularly with the movie “Three Steps Above Heaven,” a very famous movie among adolescents that shares the relationship between an aggressive conflictive boy, who is presented as highly sexually desired and successful among women, and an upper-class girl. Ana explained that, at first, she did not realize that the popular boy in the movie was aggressive—she had a different image of him—but after the interventions she recalled the movie and was able to analyze it through the lenses of the dominant coercive discourse, acknowledging that Hache, the main character, was violent:

Ana: When I came out of the cinema (this is some years ago), I saw the main character in a specific way. But after listening to you, having dialog, and with this project, I watched it again and saw it in a completely different way. Now I was not like ‘WOW, look at that guy!”

Researcher: Did you find that some flash memories about the movie have come to your mind, specific scenes from the movie, and you have recalled them and analyzed them differently? Could you give me an example?

Ana: Yes, I can now see that right from the very beginning of the movie, in what is the very first encounter between them [the boy and the girl], Hache is already insulting the girl.

Marta, another participant in the communicative focus group, shared a very similar reflection but applied to a TV series also popular among adolescents. She explained that with the interventions, she recalled the series and analyzed the affective relationship in it differently. In this case, Marta came to realize that the girl character felt attracted to a boy that despised her, while she ignored another boy who was always there for her:

Marta: I have watched a series in which the girl, at first, was dating a boy who did not treat her well, and then he left her. He kept saying to her that she was ugly, that he did not want to be with her… and she did not realize that there was another boy in her class that was always looking after her and that he liked her. Yet, in the end, she came to realize that he liked her, and she hooked up with him.

Researcher: And when you watched the series, did the project help you?

Marta: Yes

Researcher: And how did it help you in particular?

Marta: Well, with the fact that she is now dating a boy who treats her well and is good with her, and now she is happier than before when she was with another guy.

Researcher: And the project has helped you, has it given you tools for that?

Marta: Yes.

Some female adolescents shared that the language (of desire) and examples employed in the interventions, which were very close to their life experiences, induced the recall of past sexual-affective relationships that were stored in their minds as positive but, after the interventions, they came to acknowledge that such relationships were violent or toxic. Fabiola, a girl in a communicative focus group, shared this as follows, stating that such memory reconstruction changes ‘your way of choosing people’:

Sometimes you might have a memory stored in your mind, but you do not recall it… and all of a sudden, in one of the interventions of the project, a word is mentioned, and then you recall the memory. And the memory comes, and you now remember the situation differently. Now you have other resources, and you can see it as it really was. For example, if you were dating a toxic person, in the past you thought that he was a good guy, but if you thought about that memory while participating in the project, then you came to realize that he was not, and it changes your way of choosing people.

Some female adolescents shared that after the interventions they understood better the influence of the coercive dominant discourse present in the media upon their own sexual-affective preferences toward aggressive boys and they could now see better the same process occurring in some of their friends who also felt attracted to boys with violent attitudes and behaviors. In these reflections, some adolescent females showed awareness of submission to the dominant coercive discourse and discussed that the interventions in the project were useful for making some of their friends come to acknowledge the violent attitudes and behavior of boys with whom those friends had a relationship in the past; though some participants might have told the friends similar things, it was with the interventions that they recognized the toxic character of the relationship they had. Alicia shared these ideas in a communicative focus group with girls and boys:

Until 2 years ago, I always looked at the guy who had this profile [she refers to the aggressive one], right? And he was the one that attracted me, because I saw him in the series, in the movies, but then, when I saw how he treated other people. Little by little I began to change my preferences, and now I am more attracted to the boys I see that respect others, who are good people, and after these talks I have. They helped me understand why I looked at those guys in the beginning, right? And the talks help me understand what made me feel attracted to them. And now, I think about it and, honestly, those people don’t, I don’t like them. And I also have friends who, until recently, they also liked that profile of guy, and one even had a toxic relationship because of that, and, as much as I told her “he is treating you like this,” “that is so with you, that you don’t see what he is doing to you, that you are changing for him,” she did not listen to me until after these talks she also realized what had happened to her. And she told me, “Hey, you were right.” (Mixed communicative focus group, EHS1).

### New Cognitive Tools to Better Analyze Their Own and Others’ Sexual-Affective Thinking, Emotions, and Behaviors, Supporting the Rejection of Violent Intimate Relationships

The interventions provided participants with knowledge/cognitive tools that they are using to observe and better understand the sexual-affective realm of their present life in ways that can prevent gender violence victimization. Particularly, the data show that the scientific knowledge gained in the project has favored thinking about and better identification of their own sexual-affective thinking, emotions, and behaviors, along the lines of metacognition. This can be seen in this section of a communicative focus group in which participant girls acknowledge that the project has made them more conscious about the importance of attraction patterns in relation to gender violence and to see themselves as influenced by the dominant coercive discourse, for example, in realizing that some of them pay more attention to jerk boys than to egalitarian ones. In Marta’s words:

Marta: From the beginning, we didn’t see it as such a serious issue, but as you have given us examples that you put and all that. well. we have thought.

Researcher: What have you thought?

Marta: To decide who to be with.

Researcher: To decide who to be with… Can you specify a little more? Decide differently?

Marta: Yes. Changing the idea that we had, that it’s not always the jerk boy or the cool boy that is the… the ‘ideal’ guy, you know? We look more at ‘jerk’ guys, but there are guys who are good, and we don’t look at them.

Researcher: Has the project made you see this more clearly?

Marta: Yes. (Girls communicative focus group, EHS1).

There was also significant agreement among female and male participants regarding now being more equipped to discriminate between boys who fall into dominant traditional masculinities and can perpetuate violence and those who do not. The adolescents explained that the project can benefit them and adolescent girls who may feel attracted to violent boys as a result of socialization into the dominant coercive discourse so that such girls can use the knowledge shared over the seven interventions to analyze and understand the motivations behind their sexual-affective feelings, thoughts, and behaviors and redirect them toward healthier and more passionate horizons if they freely choose to do so. As Carol and Raquel explained in a communicative focus group, the interventions helped both of them better identify boys who represent a model of masculinity linked to violence, for example, by paying attention to how they treat other women; they also stated that all the knowledge gained in the project can help other girls who have felt attracted to boys with aggressive attitudes and behaviors by, first of all, being honest in acknowledging their sexual-affective preferences for such males:

Carol: Well, if there is a girl that likes the guys that treat the girls bad, this [the program of interventions] will help her out as she would realize.

Raquel: For us, it [the project] has been very positive as we have reinforced our perspectives. (…) And it has helped with how to differentiate a boy that is cocky from other ones.

Researcher: And which are the tools that you consider that the project has given you in order to differentiate that? Are there things that you focus on now in order to differentiate the nice boys from the ‘jerk’ ones?

Carol: Yes, how they treat the other ones. The way they treat other girls, and the people that are around them… if they feel superior to us or if they believe in equality. (Girls communicative focus group, EHS3).

It was common in the interviews and in the focus groups that female adolescents mostly affirmed that now, when they are on the street by themselves and see a male with aggressive attitudes and behaviors, they bring the project to their minds, think about the knowledge gained in it and quickly make the hypothesis that such male may be “jerk.” As Fatima put it: “Now, when I see a jerk guy on the street, I think of the MEMO4LOVE project.” Or, as Patricia shared: “When I see a boy like the ones you have described in the project, then you say, ‘this one could be one of those with toxic behaviors.”’

Also, the analytical tools gained in the project are not limited to individual use; the interventions have promoted new dialogs between participants and their friends both inside the high school and outside. In those dialogs, the participants bring their new learnings to analyze sexual-affective relationships and behaviors and, again, to discriminate between violent and non-violent males. In a communicative focus group with girls, some explained that when they are together talking about boys sexually, someone usually brings the project into the conversation: “Hey, let’s see what we say. Remember about the MEMO4LOVE project.”

This use of the knowledge gained in the project to analyze reality through new scientific lenses, being more active and better in discriminating between dominant and non-dominant masculinities, occurred among females and among boys. For the case of egalitarian boys, this had a very relevant meaning; the project made them feel more confident about their identification of aggressive boys and reaffirmed them in their egalitarian behavior, making them more straightforward in raising their voices in peer groups to empty violent guys of attractiveness. In a mixed communicative focus group, Carlos explained this:

Carlos: Well, outside the high school, we go with diverse people, and there is always someone new that we meet. And before, we saw that person and we said: ‘this guy is amazing, he is very cool’ and all that. But now, we see and say: ‘that guy is an idiot, he is just doing that to get our attention.’ That is something [he refers to the behavior of a male with an aggressive attitude] that, at least, my friend and I don’t accept entirely. Yet in order to avoid that, they tell us in the group that we are a pain, we shut down and that’s it. But if at some point that person crosses the red line, then we talk.

Researcher: The project, then, has helped you to be more confident to speak your thoughts, like ‘this person is not cool, he is an idiot’?

Carlos: At least, now I say it right away.

The interventions also helped participants not normalize violence within intimate relationships. Participants learned that violence is not inevitable and consubstantial to relationships but that it can be avoided and prevented. As Gemma explained in one of the mixed communicative focus groups, the interventions have helped her to reject violent and toxic intimate relationships as a first step toward not being involved in future toxic relationships, which is something that after the interventions, Gemma realized can be prevented:

Look, right now, for example, I just broke up with him (…) The memory of the relationship doesn’t bother me when I talk about it. What is useful to me is to have it as a reference for what I no longer want to happen in a relationship. And it’s like. I explained it to her [looks at a friend], I take that [the memory of the violent relationship] as a reference for something I don’t want. I don’t want it to happen again, it’s like what you just said, that suddenly you see that it is inevitable, no, that is, you have been in a toxic relationship but, uh, not all relationships have to be toxic. […] I do think, come on, it’s not inevitable!

Also, the knowledge gained through the interventions in the project makes participants feel more confident in relation to their prospective sexual affective thoughts, behaviors, and decisions. Carol explained this in a communicative focus group with girls; she said that the cognitive resources learned in the project will help her both in the case of starting dating an aggressive boy as lenses to analyze the boy and the relationship and not to engage in a relationship with a popular boy who has violent attitudes and behaviors:

The fact of having resources makes you feel more secure. If I start dating a boy who is ‘bad,’ I would have resources obtained after having done this project, to remember what we talked about and I would think, reflect… and if it is my boyfriend, I would rather date somebody who is not popular than suffering for dating somebody who is ‘popular’ (Girls communicative focus group, EHS3).

### Change in Preferences and Attraction: Greater Rejection of Violent Men and Enhanced Attraction Toward Egalitarian Masculinities

The interventions favored a change in female adolescents’ perceptions of violent boys and toxic relationships, transforming the way they perceive those boys, increasing rejection of violence and supporting a positive change in their preferences for different types of men. In the following quote, Camila and Amina show that, after the interventions, they are more aware of the presence and consequences of violent intimate relationships, increasing their rejection of aggressive boys who are now perceived as less attractive than before:

Researcher: Do you think that the interventions have helped you to change that idea?

Camila: Yes

Researcher: In what way? How? Explain to me a little how you saw it before and how you see it now, after the interventions.

Camila: (…) Seeing it in another way, you stop liking that kind of boy because of all the things you’ve seen [in the program] and what can happen, and then it’s like different, and it changes the way you think and how you see that person.

Amina: The same as her. (Mixed communicative focus group, EHS2).

Rejecting violence and toxic intimate relationships has been one of the most successful results of the interventions, which followed a change among adolescent girls in their sexual preferences for different types of men. As Nuria started explaining, the interventions promoted participant girls to move from paying sexual attention to aggressive boys, the ‘jerk’ and ‘jerk’ ones, who were, after the interventions, less attractive for these girls, and to start paying attention to egalitarian boys:

Researcher: Do you think that, as a result of the project, it is like you see more, or do you pay more attention to boys who treat women well?

Several girls: Yes.

Researcher: Well, explain.

Nuria: (.) At one time, I liked the jerk guys. And then when you mature, you realize. And from the project, I have realized even more, I have opened my eyes more. (…) In my case, boys who I saw as handsome, now I see them in person, and I see them as they really are, and I don’t like them anymore.

Researcher: And does the project have to do with that?

Nuria: Yes. (Girls communicative focus group, EHS3).

Along these lines, Nuria openly shared the deep change in her sexual preferences and desires for different types of men supported by the project. Thanks to the interventions, Nuria was able to identify a boy who behaved in a toxic way and to distinguish him from another boy who was egalitarian. Yet Nuria went a step further and started considering the egalitarian guy as more desirable and attractive than the violent one. In her own words, the nice guy was now “every time higher in the ranking.” The interventions provided Nuria not only with cognitive tools for identifying and analyzing toxic behaviors and attitudes but favored a change in her sexual preferences in favor of liking egalitarian men:

Nuria: There is a coach who is a little jerk, he is very handsome, very attractive. At first, he caught my attention, but as I saw that he was a little ‘jerk,’ I stopped liking him. And then, on the other hand, there is my brother’s coach who is not…, he behaves very well with children and he speaks to me like super good, he has been kind. And then I realized that I liked him more, and being with men who treat children well, if they treat a child, who is innocent, well, then they will treat other people well too.

Researcher: Is it like, then, you started seeing the nice boy with different eyes?

Nuria: I already saw him [the ‘jerk’ boy] like that, but I saw the other one [the egalitarian one] differently. And now it’s like he [the ‘jerk’ boy] swapped positions in the ranking. Now is like I can’t bear him. And the other one [the egalitarian boy] is every time higher in the ranking. (Girls communicative focus group, EHS3).

Importantly, the transformative change in the desire and sexual preferences for different types of men reported by some adolescents also impacted, in some cases, their decisions regarding their sexual-affective relationships, if they had any. Some of the adolescents started dating egalitarian boys that, before the intervention, they would never have paid attention to and who were not considered by the adolescents to be as attractive as the “jerk” boys:

Researcher: Have you started paying more attention to boys that you didn’t notice before?

Several girls: Yes!

Researcher: Examples?

Ana: I’m dating a guy who, a while ago, I would never have thought I would date. (Girls communicative focus group, EHS1).

### Taking a Stand and Giving Better Advice to Friends, Peers, and Others in the Community

The impact of the project went beyond adolescent participants and beyond the high schools where the research was conducted. Some of the adolescents went a step further and used the cognitive tools learned in the project to help their friends and other community members in reflecting upon their *sexual preferences* for different types of men, about the *quality of their intimate relationships* and the different *effects of violence and toxic relationships on the brain and overall health*. All of these were contents of the interventions of the project that were used by adolescents to give better advice to friends and others in the community. As Neus explained, the project helped her to take a stand against violence and give better advice to one of her friends who was in a toxic intimate relationship:

Neus: For example, when a friend tells me that she is with a boy and I know the boy is a ‘jerk’…, before, I wouldn’t have told her anything. Now, however, I would encourage her to split up with him because he is not suitable to her and, taking into account the topics we have spoken about, I can give her more and better advice.

Researcher: Do you feel that you give more and better advice than before? Have you experienced during these months having given advice to a friend?

[Neus noods].

Researcher: [to the rest of the group] Have you also experienced giving better advice?

Ana: Or even giving them to yourself. (Girls communicative focus group, EHS1).

Another adolescent, Teresa, who participated in a mixed communicative focus group explained that during the project she started talking with one of her friends; they dialoged and helped each other, sharing advice to improve their intimate relationships based on the knowledge gained in the interventions. This made it possible to achieve a shift in the relationships that they were having by that time, avoiding such relationships becoming toxic, as they had experienced in the past:

Teresa: We helped each other using the sessions of the MEMO4LOVE Project and our previous experiences.

Researcher: Nice, and how?

Teresa: For example, if there was something in my relationship or in her relationship that I did not like or that she saw was not right, then, taking into account what we have seen here and what we knew from before, we have managed to make the relationships of both of us more beautiful.

Researcher: That is very nice. Have those changes in the relationships happened?

Teresa: Yes.

Researcher: And how has that made you feel?

Teresa: Better

Researcher: And is the relationship better now?

Teresa: Yes.

Researcher: Could you tell me which ideas or which interventions from the project have been more useful in making that happen?

Teresa: The one that had most impact on us was the one related to health. We knew that having a toxic relationship was bad, but we didn’t know that it could affect your neurons and all that. That is so heavy! Given that toxic relationships affect that much… and because we both had already experienced certain things, then we started thinking and then talking, and we said that if we continued the way our relationships were going, it was going to be like past relationships, toxic, and we said that we did not want that. (Mixed communicative focus group, EHS1).

**FIGURE 2:**
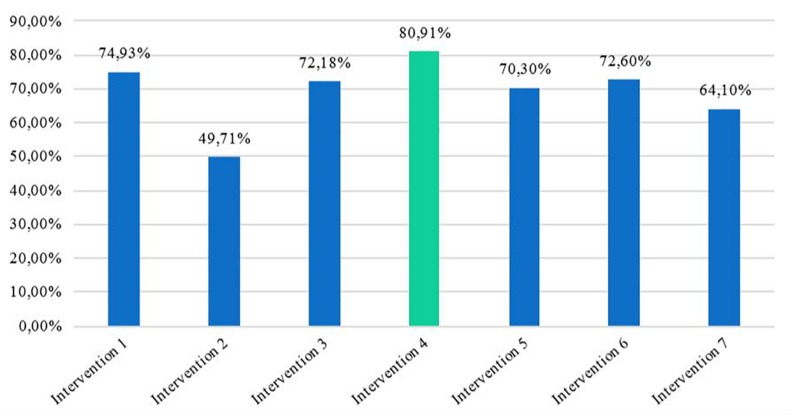
Impact of the aggregated positive change in the three dimensions across the seven interventions.

In this regard, participants agreed that intervention number four, which focused on the impact of sexual-affective violent relationships on the brain and overall health, had a great impact on raising their awareness about the serious consequences of violent relationships and even made some change their sexual-affective decisions. Quantitative data from the surveys in which participants assessed every intervention also supports this participant perception. As shown in Figure 2, all seven interventions were highly valued (five out of seven, more than 70%) by the adolescents on the three dimensions measured in the surveys: the interventions aided change in adolescents’ thinking about the topic, improved their understanding of the participants’ own and other people’s behavior and feelings, and improved the adolescents’ ability to help others in the realm of sexual-affective relationships. Nonetheless, it was intervention number four that generated the most positive change, reaching almost 81% of positive evaluation in all of the three dimensions measured together.

**FIGURE 3:**
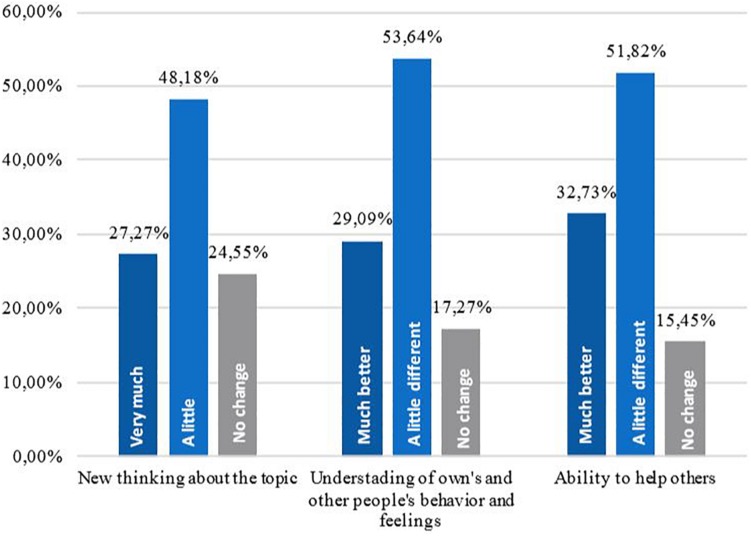
Impact of Intervention number 4 in the three dimensions measured.

As shown in Figure 3, focusing on each of the dimensions measured on intervention 4, on average, and with little deviation, 30% of participants stated that intervention 4 generated a very positive change in the three dimensions. 27.27% of the participants declared that intervention 4 contributed very much to their having new thinking about the impact of violent intimate relationships on health. 29.09% of participants indicated that this intervention helped them understanding much better their own and other people’s sexual-affective behavior and feelings. Lastly, 32.73% of participants pointed out that the 4^*th*^ intervention increased their ability to help others regarding sexual-affective relationships far more than the others.

The qualitative data from the communicative focus groups are coherent and reinforce the positive change shown in this quantitative data. For some participants, getting to know the scientific evidence on the impact of violent intimate relationships, either sporadic or stable, on the brain left many adolescents thoughtful and motivated to change their behavior, actively reject violence, and share this knowledge with their friends and other people in the community to help them. As the following quote shows, when the health intervention ended, Carol thought about her mother’s friend, who had many health problems that were not to be expected considering the woman’s age, 43. That day, after leaving school, Carol went to find her mother’s friend to explain what she had learned in intervention 4. Carol had increased her awareness about the damage of toxic relationships on the brain and overall mental and physical health and brought that knowledge to another woman from the community to caution her about the type of relationships that she had. Carol had the appropriate cognitive tools to think critically about the health condition of her mother’s friend who was involved in a toxic relationship. The information Carol shared, particularly the knowledge gained on the *Telomere Effect* (Blackburn and Epel, 2017), the effect of toxic stress on shortening telomeres, made that woman aware of the effects of violence and toxic relationships on her own health:

Carol: Each time that I see this woman that I know, who has a toxic relationship, she says that she has pain in her knee, and she is not that old to suffer that pain.

Researcher: How old is she?

Carol: 43. So when the session finished, I thought about her and told her that the pain she suffers could be due to that effect [the *Telomere effect*].

Researcher: And did you speak to her? How was it?

Carol: She was very stressed.

Researcher: And do you think that the fact of you going there, telling her that and showing her the link between toxic relationships and illness helped her? If so, how did you notice that?

Carol: Yes, by her face, I could see that she had not realized it before. (Girls communicative focus group, EHS3).

This case exemplifies that the impact of interventions transcended the participants themselves. In the case of intervention 4, the adolescents used knowledge of the impact of intimate partner violence/teen dating violence on health to give advice to their friends, to raise their awareness about the serious consequences of violence in sporadic or stable relationships, and, from there, to encourage their friends to leave toxic relationships. Crucially, some of the participants’ friends left abusive relationships or modified their preferences for different types of men after receiving such informed advice. Teresa shared such a case. One of Teresa’s friends was in a toxic intimate relationship, and Teresa took her aside and talked to her using the knowledge gained in the project. One of the most persuasive arguments that Teresa used with her friend was when she provided insights from intervention 4 on health, particularly about the neuronal death and loss of synaptic connections following toxic relationships. This was the determinant for Teresa’s friend, who decided to break up with the boy she was dating:

Teresa: I knew that a friend had a bad relationship, and I spoke to her and explained what she could do. (…) And it helped her a lot because she ended up splitting with him.

Researcher: Was she the one finishing the relationship?

Teresa: She was the one splitting up.

Researcher: She split up? -the girl nods-, after having spoken with you? -the girl nods- and which arguments did you use? Which ideas did you pick up from the project?

Teresa: I always see her crying a lot, she was always angry, depressed, and I told her, “Do not continue with this because you are hurting yourself.” I told her about the neurons [she refers to the telomere effect and the neural death associated with the stress resulting from violence], and she told me that she didn’t know that and appreciated the fact that I had told her, and she gave me a hug, and then she split up with him. (Mixed communicative focus group, EHS1).

## Discussion

The fifth sustainable development goal of the United Nations refers to Gender Equality. Achieving such a goal involves tackling violence against women around the world (United Nations Statistics Division, 2015), with a particular concern for girls who are victims of sexual violence. Thus, one of goal 5’s targets is to “Eliminate all forms of violence against all women and girls in the public and private spheres, including trafficking and sexual and other types of exploitation.” In making this social target real, the scientific community plays a central role, investigating which interventions can be most effective in preventing gender violence and in responding to it most successfully, as well as in informing the design of preventive programs. Psychology has played a fundamental role in providing scientific evidence on the causes and consequences for gender violence victimization and in noting the developmental importance of starting preventive work at the adolescence stage (Blattner et al., 2015; Lundgren and Amin, 2015) and the need to count on schools as sites at which to implement prevention programs (Ozer, 2006; Lundgren and Amin, 2015). Upon this body of research, some researchers in psychology and in other social and health sciences have designed preventive programs, some of which have demonstrated ability to reduce the risk for gender-based violence victimization and revictimization and to decrease victimization itself (Foshee et al., 2005; Wolfe et al., 2009; Taylor et al., 2013, 2015).

The MEMO4LOVE Project adds to this body of knowledge and evidence-based interventions. However, it does so from a new interdisciplinary research perspective, that of preventive socialization of gender violence, which starts from the evidence on the strong influence of the dominant coercive discourse (link between attraction and violence) in the sexual-affective learning of adolescents and its role in gender violence among teenagers (Gómez, 2015; Puigvert et al., 2019). Grounded on such research, the project involved the implementation of seven interventions of preventive socialization of gender violence in secondary school classrooms, and it was addressed to 15- and 16-year-old adolescents. The interventions focused on seven different topics, but all were dimensions of the influence of the dominant coercive discourse in adolescents’ sexual-affective life, accompanied by egalitarian dialogs with the adolescents on how to break free from such discourse and thus avoid violence in sporadic or stable sexual-affective relationships.

The analysis of the data, mostly the adolescents’ perceptions of the impact of the interventions, has shown that the project has produced benefits in attitudes toward violence, relationships, skills, and emotions that the scientific literature in the field has noted to be instrumental in preventing and reducing gender violence in adolescence (Leen et al., 2013; Gómez, 2015; Miller et al., 2015). The adolescents shared that the project has helped them raise awareness about violence in intimate relationships, be better equipped to analyze sexual-affective relationships more critically, reduce attraction toward violent dominant traditional masculinities, take a stand in the face of violent attitudes and behaviors, and help others to prevent their gender-based violence victimization.

Firstly, the adolescents who took part in these interventions reported developing critical consciousness regarding the presence of the dominant coercive discourse in society and in their lives. This happened both via recalling sexual-affective autobiographical memories and/or by analyzing present experiences of their own and of others where the dominant coercive discourse manifests. Other participants shared memories of the presence of the dominant coercive discourse in the peer group, acknowledging that peers can coerce others to like boys with aggressive attitudes and behaviors and even to have a relationship with them. This is an important finding. Some studies (O’Sullivan and Meyer-Bahlburg, 2003; Collins et al., 2009) have pointed out the pressure that the female peer group can exercise upon a girl to have a relationship with a boy. If such pressure is aligned with the dominant coercive discourse, as some adolescents shared that it was in our study, then this is one more reason for prevention programs to focus on the peer group together. The greater the transformation of peer interactions, the greater the prevention of gender-based violence victimization. This is a potential social impact that derives from the study.

Also, generally, adolescents agreed that the interventions made them more able to “see” the dominant coercive discourse illustrated in the movies, singers, famous TV series, etc., that they follow. This consciousness is a first step in the potential process of weakening the connection between males with aggressive attitudes and behaviors and an emotional response of attraction, and this debilitation could protect this youth from gender-based violence victimization (Gómez, 2015). As the adolescent participants shared, the examples of the dominant coercive discourse presented in the interventions made them aware of the influence that this discourse had on them; the interventions generated a crucial opportunity for the adolescents to take a stand in the face of the dominant coercive discourse, as active independent people who decide not to subjugate their sexuality to it or stop doing so. This attentional (raising awareness) work among adolescents in relation to their everyday life experiences is important, given the very widespread teaching of this discourse via TV series, songs, movies, etc.

Secondly, as Aiello et al. (2018) have indicated, it is necessary that intervention strategies in education provide adolescents with tools that enable them to be more critical about violence in society. The program presented in this study showed effectiveness in reaching this goal. The adolescents shared that the interventions provided them with cognitive tools to better analyze their own and others’ sexual-affective thinking, emotions, and behaviors in ways that favor the rejection of violent males and violent sexual-affective relationships. Some participants affirmed that these tools helped them to better analyze and understand their past and present sexual relationships, understanding that violence can be avoided and prevented in relationships. This supported more profound thinking in the adolescents about what they wanted their intimate relationships to be like in the future, favoring the rejection of violent sexual-affective relationships. All this might indicate changes in adolescents’ attitudes toward violence, one of the risk factors for gender-based violence victimization (Leen et al., 2013).

A third potential impact of the program, as shared by the adolescents, is particularly relevant: change in preferences and attraction, with greater rejection of violent men and enhanced attraction toward egalitarian masculinities. The key feature of the dominant coercive discourse is that it mostly operates at the emotional level. Consequently, it is significant to be aware of the dominant coercive discourse, but what is even more fundamental is to change the response of attraction toward men with violent attitudes and behaviors; that is what will make a difference in terms of preventing gender-based violence victimization. Puigvert and Flecha (2018) refer to *coerced preferences* as those that are imposed by the dominant coercive discourse and then internalized by the individual, something that has been evidenced to play a crucial role in engaging in violent sporadic relationships (Puigvert et al., 2019). Our results shed light on some adolescents coming to revise their coerced preferences along the course of the interventions in the project. This proves that affective models of attraction can be changed through simple social experiences, as not only psychology and other social sciences (Gómez, 2015) but also neuroscience have shown when differentiating between emotions and feelings (Kandel et al., 2013). This emotional plasticity reiterates the ability of certain educational interventions to give adolescents the opportunity to revise and transform their affective models of attraction, moving from attraction to men with violent attitudes and behaviors to egalitarian masculinities. Other interventions within the field of preventive socialization of gender violence, such as the *Heroes’ Club 0 Violence*, had already been shown to be effective in the endeavor of emptying violence from attractiveness since early childhood (European Commission, 2015). Furthermore, moving one step further, from feeling to action, some adolescent girls declared that they ended toxic relationships and started dating egalitarian boys, acknowledging that the interventions helped them in making this change. This was very significant for some, who stated that they were now paying attention to or dating egalitarian boys whom they had never thought of before in sexual terms.

Leen et al. (2013) indicated that achieving behavioral changes in relationships, skills, and attitudes seems to be a factor that can help with the sustainability of changes induced by interventions in this area. All of the results discussed above involve skills, attitudes, and relationships, and so they might contribute to the potential sustainability of the new learnings in participant adolescents in the project. However, this is something that should be investigated by following the same subjects and measuring (quantitively and qualitatively) again in the near future and beyond. Another important factor that may help in sustainability is the promotion of healthy relationships (Miller et al., 2015). Young people have to know which are the characteristics that define a healthy and passionate relationship, but they also have to feel secure enough to defend this type of relation. Because of the force of the coercive dominant discourse in some peer groups, it can be difficult for some adolescents not to support their friends’ discourse. Bystander behavior can help create a secure environment where adolescents feel free enough to defend a different discourse that prioritizes and defends healthy relationships. Programs such as Take Care (Sargent et al., 2017) or the intervention carried out by Miller et al. (2013) with athletic coaches show their effectiveness by improving bystander behavior in the face of gender violence. Our study makes a complementary contribution that can also contribute to transforming the hostile environments sometimes created by peers in high schools (Fineran and Bennett, 1999). The results indicated that the set of interventions gave more confidence to adolescents who had already rejected the dominant coercive discourse and the violent relationships that it imposes and reaffirmed these adolescents’ stand. This is a key finding, as research has shown that peer influence is a risk factor that could influence the perpetration of gender violence (Leen et al., 2013). It is vital that young people who already have an idea about what a healthy and passionate sexual and affective relationship is feel confident enough to defend their position within their peer group. Participant adolescents said that the project gave them more security to trust their own choices, making them free to make decisions according to what makes them feel better and not being conditioned by social pressure and the opinions of their peers. Some adolescent boys shared that the intervention gave them enough knowledge and security to raise their voices in front of the peer group and dare to give a different opinion on men with violent attitudes and behaviors, rejecting the attractiveness of such men. This is a very substantial result that constitutes another area for further exploration, as this position might help other members in the group not to submit to the dominant coercive discourse imposed by some group ‘leaders.’ Likewise, such a brave stand can constitute one more way to build secure environments where adolescents can feel emotionally safe.

Prior research in psychology has shown that programs for the prevention of violence that foster communication contribute to building a sense of community that provides good results, for example, the Expect Respect program (Ball et al., 2009) and the Healthy Relationship Campaign (Guillot-Wright et al., 2018). The importance of sharing ideas, talking, and reflecting with friends or other people was confirmed in our study, while shedding new light. Our findings show that the benefits obtained by the development of critical consciousness and learning of new cognitive tools in the participants impacted not only their personal lives but sometimes had impacts that also extended to friends and other people in the community. Some participants spontaneously decided to use the knowledge gained in the interventions to improve the intimate relationships of their friends, relatives, and community members who were experiencing a toxic relationship. Some adolescents even explained that thanks to sharing such knowledge with the motivation of helping others to escape violence, some people around them – who were not participants in the interventions – became more conscious, left abusive relationships, or modified their sexual preferences for different types of men. This is an important potential social impact of the program that deserves attention. Among the knowledge shared with the adolescents, it was the scientific information on the damaging consequences of violent relationships on the brain and overall health that seemed to have moved them more to reach out diverse victims to help them. This proves the ability of adolescents and youth to not only understand sophisticated scientific knowledge on violent sexual-affective relationships but also use such knowledge to improve their lives and those of their loved ones. The discussion of original scientific knowledge with adolescents in gender violence prevention programs seems a line with potential social impact that should be further explored, which also makes real article 27 of the Universal Declaration of Human Rights United Nations (1948), which establishes all humans’ right to access scientific knowledge and to participate in it.

There are limitations to the research reported. The first refers to the stability of change. The measures employed and reported here (focus groups, interviews, and assessment surveys) to evaluate the perceived impact of the project on the adolescents were conducted in months 6 and 7 of the intervention program; that is, after intervention 6 and the next month. To analyze the permanence of change, this measurement should be repeated at various times several months after the last intervention. Such data, together with the other quantitative measures (standardized tests on attitudes and memories) employed in the MEMO4LOVE research project, could reveal more about the power of the program of seven interventions in supporting adolescents’ breaking free from the dominant coercive discourse, mentally and affectively, in the mid and long term. Persistent changes in attitudes, behaviors, and feelings are necessary to better prevent future gender-based violence victimization. This is an area for future research. A second limitation might be the self-reported nature of the data. It could be argued that the results are affected by social desirability (McDonald and Hirt, 1997). Although we collected data through validated quantitative instruments, such as the IPVAS scale on gender violence attitudes, for examining the potential social impact of the project, we decided to include the voices of adolescents. We did this for two reasons. One, because this interpretative approach is scarce in the area when assessing the impact of prevention programs, and, two, because existing scales measuring gender-based violence victimization and gender violence attitudes do not contemplate the most common types of first sexual-affective relationships among adolescents, which are sporadic, and where much violence occurs (Puigvert et al., 2019). The MEMO4LOVE interventions included many reflections on violence in sporadic sexual relationships, so instruments that allowed the examination of this reality (and changes in it influenced by the project, if any) were needed. The qualitative measures made this possible. Also, though not a limitation, there is a greater number of quotes from female adolescents in this manuscript. This has to do with the fact that the project starts from the evidence that the prevalence of gender-based violence victimization is much higher among women. Therefore, the changes reported in the manuscript are of particular significance among female participants.

In all, the study reported here illustrates the important social impact that psychology, together with other social sciences, can have in the prevention of gender-based violence victimization among adolescents and youth. Psychological research has long informed programs for the prevention of and response to gender violence in adolescence (De La Rue et al., 2017). In this manuscript, we have added a new contribution to this body of evidence-based programs: a set of seven interventions grounded in research results from the field of preventive socialization of gender violence that focuses on raising awareness about the presence of the dominant coercive discourse in adolescents’ sexual-affective life, thoughts, and feelings; however, mostly, the interventions emphasized providing tools with which the adolescents could revise these dimensions to empty violence of attractiveness and ultimately to prevent gender-based violence victimization. The results provide information on the potential social impact of the interventions, echoing Santiago Ramón y Cajal’s (1989) metaphor about plasticity. The outcomes of the project unveil the ability of every adolescent to be the architect of her or his own brain by means of freely choosing to break free from the dominant coercive discourse. In doing so, adolescents can improve not only their own lives but also those of their communities.

## Data Availability Statement

The datasets generated for this study are available on request to the corresponding author.

## Ethics Statement

The studies involving human participants were reviewed and fully approved by the Research Ethics Committee of the Virgen de la Macarena and Virgen del Rocío Hospitals (Government of Andalusia, Spain), as well as by the Ethics Committee at Universidad Loyola Andalucía (Spain). Written informed consent to participate in this study was provided by the participants’ legal guardian/next of kin. Written informed consent was obtained from the minor(s)’ legal guardian/next of kin for the publication of any potentially identifiable images or data included in this article. Written informed assent was also obtained from all participant minors.

## Author Contributions

SR-P conducted the research and investigation process. LU, GM, and NG-F participated in the data analysis, with a particular focus on the potential social impact of the research project. SR-P, LU, GM, and NG-F interpreted the data for the work and contributed to the formal analyses and discussion of the data and revised it critically for important intellectual content and approved the submitted version. SR-P, LU, and GM drafted the manuscript.

## Conflict of Interest

The authors declare that the research was conducted in the absence of any commercial or financial relationships that could be construed as a potential conflict of interest.
